# What is the effect of mobile phone text message reminders on medication adherence among adult type 2 diabetes mellitus patients: a systematic review and meta-analysis of randomized controlled trials

**DOI:** 10.1186/s12902-023-01268-8

**Published:** 2023-01-19

**Authors:** Abebe Muche Belete, Belete Negese Gemeda, Tadesse Yirga Akalu, Yared Asmare Aynalem, Wondimeneh Shibabaw Shiferaw

**Affiliations:** 1 Department of Biomedical Science, Debre Berhan University, P. O. Box 454, Debre Berhan, Ethiopia; 2 Department of Nursing, Debre Berhan University, Debre Berhan, Ethiopia; 3 Department of Nursing, Debre Markos University, Debre Markos, Ethiopia

**Keywords:** Mobile phone text message, Type 2 diabetes, Medication adherence

## Abstract

**Background:**

Globally, type 2 diabetes has become increasing. As little is known about the effect of educational intervention on this population, this systematic review and meta-analysis evaluated the effectiveness of mobile phone text message reminders versus usual care to improve medication adherence among type 2 diabetes mellitus patients.

**Methods:**

PubMed, Google Scholar, Cochrane Library, Scopus, and African Journals Online, were searched. A random-effects model was employed to estimate combined effect sizes. Subgroup analyses were employed to investigate possible sources of heterogeneity between studies. The overall certainty of the evidence was evaluated using the Grading of Recommendations Assessment, Development, and Evaluation approach.

**Results:**

A total of 9 trials with 1,121 participants were included in the review. The pooled estimated impact of mobile phone text message reminders on medication adherence was (SMD: 0.36; 95%CI; 0.14, 0.59) compared to usual care groups among patients with type 2 diabetes mellitus. In addition, subgroup analyses revealed greater medication adherence levels in those studies with intervention durations of more than six months and with self-report/refill adherence scale measurement (SMD: 0.21; 95%CI: 0.02, 0.40) and (SMD: 0.45; 95%CI: 0.22, 0.68), respectively.

**Conclusion:**

Mobile phone text messages can potentially lead to improved medication adherence levels in patients with Type 2 diabetes despite heterogeneity across the studies. Therefore, mobile phone text messaging when delivered in addition to usual care, have the potential to produce significant improvements in medication adherence.

**Supplementary Information:**

The online version contains supplementary material available at 10.1186/s12902-023-01268-8.

## Introduction

Diabetes mellitus (DM) has become a widespread non-communicable health problem, resulting in significant morbidity and mortality [1]. About 422 million adults worldwide live with DM [2]. The International Diabetes Federation estimates that about 629 million people will be affected by 2045 [3]. About 80% of the affected people live in low-income countries[4]. In particular, type 2 diabetes (T2DM) accounts for more than 90% of all diabetes cases [5]. The increased burden is due to multiple risk factors such as physical inactivity, obesity, unhealthy diet, physical inactivity, family history, age [6, 7], and oxidative stress [8].

Medication non‐adherence is one of modern medicine's significant health challenges; poor medication adherence is associated with increased morbidity, mortality, and healthcare costs. [9]. Improving adherence to medications for T2DM patients would help to maximize the clinical benefits for the wider population [10]. There is, therefore, considerable scope for increasing adherence to prescribed medicine, thereby reducing morbidity, mortality, and healthcare costs.

Telemedicine can be a strategy for closer monitoring and intervention to achieve better metabolic control and help in the global care of individuals with concomitant chronic diseases [11]. Text messaging is increasingly used in medical practice to remind patients of clinic appointments, to report test results, and to adjust treatment doses [12]. In addition, text messages can help remind patients to take their medications, recognize who is not taking them and why, if any, and provide appropriate advice in such cases [13].

The use of text messages is vital for improving diabetes treatment adherence and glycemic control [14–18]. Therefore, mobile phone‐based interventions are of particular interest, given their low cost and potential for widespread delivery. However, no systematic review has specifically examined the effect of mobile phone text message interventions on adherence to medications for T2DM. Hence, the present review and meta-analysis aimed to evaluate the effect of mobile phone text message interventions on medication adherence in patients with T2DM.

## Methods

### Protocol and registration

The study protocol was registered on Prospero (www.crd.york.ac.uk/PROSPERO/) as recommended by the PRISMA statement [19] with the number (CRD42021285017).

### Search strategy and data sources

Our search strategy employed the methodology of the peer review of electronic search strategies (PRESS) for systematic reviews [20]. We searched PubMed, Scopus, Google Scholar, African Journal Online, and Cochrane from January 2000 to March 4, 2022, for relevant articles (as authors are interested in up-to-date data). The search was conducted using medical subject heading (MeSH) terms and keywords: type 2 diabetes mellitus, diabetes mellitus, adult, short message system/SMS, text messaging, mobile phone text message, diabetes mellitus/drug therapy, medication adherence, health education, telemedicine, and randomized control trial. Date last searched March 4, 2022. The search was restricted to full texts, human studies, and English language publications. The entire search for PubMed is attached in Supplementary Table 1 and documented in our entry in Prospero.

### Eligibility criteria

Following the PICOS model:

### Patient, Population, or Problem

Adult (age > 18 years) patients with type 2 diabetes mellitus.

### Intervention

Mobile phone text message intervention or reminders.

**Comparison**: usual care or routine care groups of patients for type 2 diabetes mellitus.

**The outcome you would like to measure or achieve**: anti-diabetic medication adherence among type 2 diabetes mellitus patients.

### Type of Study you want to find

A randomized control trial study (RCT) aimed at the effect of mobile phone text message intervention to improve medication adherence with quantitative results, ideally with means and standard deviations/errors reported.

### Criteria for considering articles for inclusion

All published mobile phone text message intervention RCTs to improve medication adherence among type 2 diabetes mellitus patients were considered in this review. We limited papers considered to those published from 2000 and were a whole paper was available. Papers in English were considered. The Authors included studies comparing intervention and control groups.

### Exclusion criteria

Any study not meeting the inclusion criteria or that did not report quantitative data of the relevant medication adherence score other than the RCTs study design. We excluded studies that used text messages and phone call interventions. Besides, we excluded studies where the means and standard deviations were missing for the meta-analyses.

### Study selection

Two review authors (AM and BN) independently screened titles and abstracts of the search results and discarded studies that were not applicable. However, studies that might include relevant data or information on studies were retained initially. We retrieved the full-text study reports of all potentially eligible studies, and two review authors’ (AM and BN) independently screened them for inclusion, recording the reasons for excluding ineligible studies. We resolved disagreements through discussion until we reached a consensus or, if necessary, consulted another review author (YA). We identified and excluded duplicates and collated multiple reports of the same study so that each study, rather than each report, was the unit of interest in the review. Cohen's κappa was run to determine whether the two authors' judgments agreed on whether studies were included or excluded from the review. There was moderate agreement between the two authors’ judgments, κ = 0.809 (95% CI, 0.583 to 0.961), *p* = 0.001.

### Data extraction and management

We generated a data extraction form and pilot-tested it. After verification, two review authors (AM and BN) independently extracted data from the included articles. Characteristics of included articles; the numbers of participants, type of participants, setting (countries and type 2 diabetes mellitus), interventions, comparisons, relevant outcomes with definitions, and information for any adjustments. Risk of bias assessment included articles such as the method used, domains assessed, and judgments. Characteristics of interventions; population (mean age, type 2 diabetes mellitus status), the form of mobile text message (amount of messaging, received reminders, motivational and supported messages, and advice on lifestyle behaviors like diets, physical activity, smoking cessation, medication, and appointment reminders), frequency, start and duration of intervention, measures of adherence to the intervention, and adherence to anti-diabetic medication.

We presented the review details and results in tables. Another two review authors (WS and TY) verified the extracted data. We resolved any discrepancies through discussion until we reached a consensus. Where any information from the reviews needed to be clarified or included, we accessed the published papers of the individual trials.

### Risk of bias in individual studies

Three review authors (WS, YA, and TY) independently assessed the risk bias of the included articles. We used the revised Cochrane risk-of-bias tool for randomized trials (RoB 2) [21], which considers random sequence generation, allocation concealment, blinding of participants and personnel, blinding of outcome assessment, incomplete outcome data, selective reporting, and other potential sources of bias. The risk of bias in the RCTs was examined using the method described in the RoB 2 tool. We categorized each domain as 'high risk,' 'low risk,' or 'some concerns' using the algorithms proposed in RoB 2. Next, we assessed the overall risk of bias. We considered a study: to be at high risk of bias when at least one domain was judged as being at high risk, to be at low risk when all domains were judged as being at low risk and to raise some concerns when at least one domain was judged to raise some concerns. However, no domains were judged as being at high risk of bias. Any disagreement was resolved through discussion and consensus, and if required, we consulted a third review author (AM).

### Subgroup analysis and investigation of heterogeneity

We first assessed the heterogeneity by visual inspection of the forest plot. Then, we quantified statistical heterogeneity using the I^2^ statistic, which describes the percentage of total variation across studies due to heterogeneity rather than sampling error [22]. Finally, we conducted a subgroup analysis to explore the possible source of heterogeneity. We classified the measure of medication adherence into the Morisky adherence scale and self-report/refill. The results were reported individually and combined with the Morisky adherence scale and self-report/refill (i.e. Morisky adherence Vs self-report/refill). Additionally, we classified based on the duration of intervention, i.e. three months, six months, and 24 months duration. Again, the results were reported individually and combing the three (i.e. three months Vs six months Vs 24 months).

### Sensitivity analysis

We performed the leave-out-one approach method.

### Assessment of publication bias

Assessment of publication bias was carried out using a funnel plot and egger’s test.

### Data synthesis

Using a random-effects model, the outcome was reported as a standardized mean difference (SMD) with a 95% confidence interval (CI). In addition, the papers' results were pooled, where data were comparable, using a meta-analysis package employing the Review Manager of the Cochrane Collaboration (RevMan 5.4, Cochrane Organization).

### Summary of the findings and quality of the evidence in included articles

We created a summary of the findings table using medication adherence as the outcome. The overall quality of the evidence was evaluated using the Grading of Recommendations Assessment, Development, and Evaluation (GRADE) approach [23]. We summarized the quality of evidence using GRADE pro-GDT Cochrane online software [24]. The GRADE quality assessment component is comprised of methodological worth, indirectness of evidence, unexplained heterogeneity, imprecision, and the probability of publication bias. The GRADE approach is focused on determining within the study risk of bias. It has four levels of quality such as high-quality evidence, moderate quality, low quality, and shallow quality.

## Results

### Results of the search

**Fig. 1:**
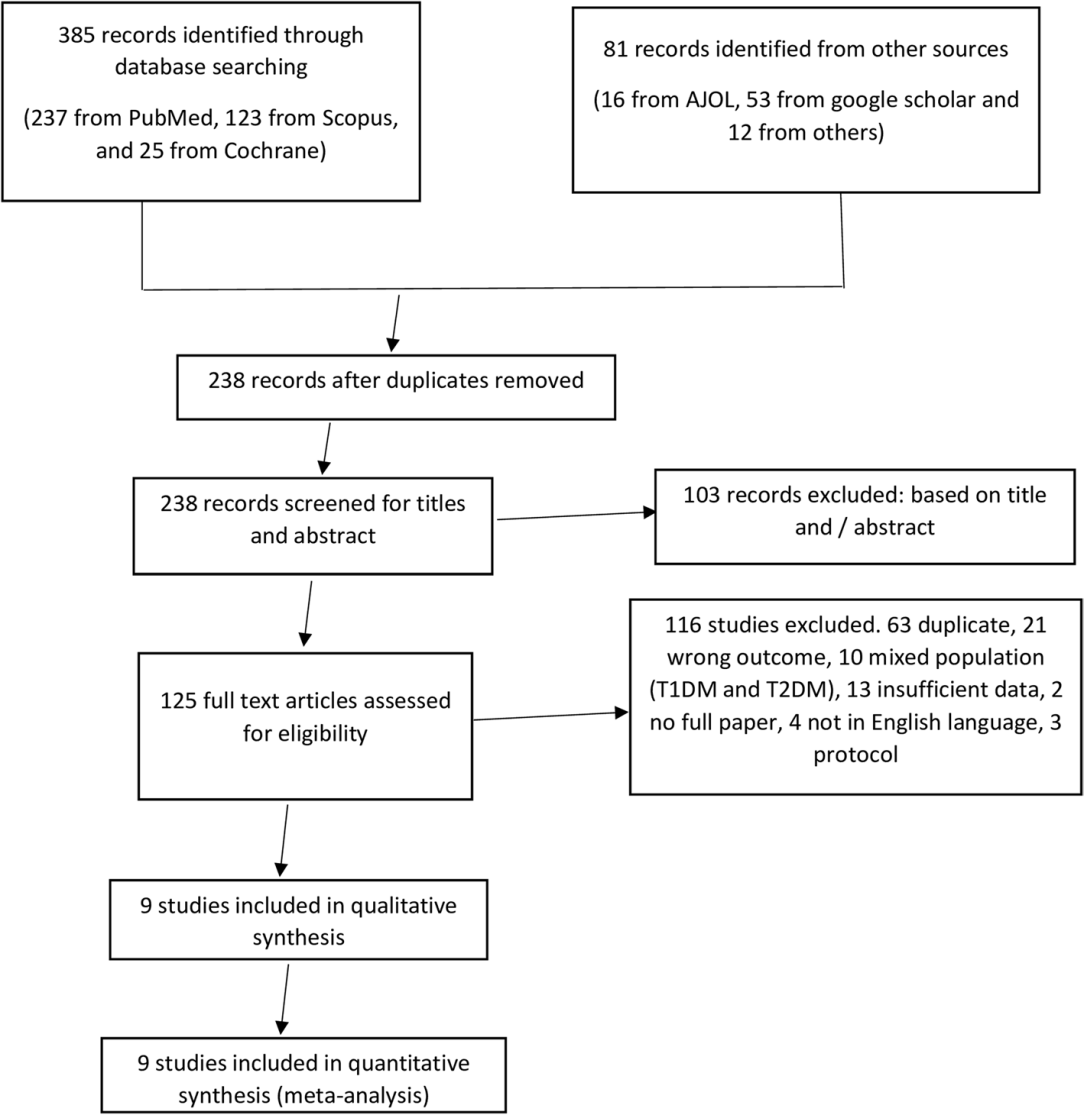
PRISMA flow diagram for study selection

After searching the databases and other sources, 238 records were identified. After screening titles and abstracts and full-text review, 9 studies were included and 228 studies were excluded for various reasons as shown in Fig. 1.

### Characteristics of the included studies

We included nine studies with a total of 1,121 participants. The study characteristics, including demographics of the study, durations of intervention, frequency of the short message system, the content of the short message system, and measurement of medication adherence, are presented in Table 1. A total of 8 studies were randomized control trials [15–17, 25–29], and one study was quasi-experimental design [30]. In addition, a limited number of studies used the Trans-Theoretical Model [26] and behaviour change theories [27] as an education platform.

**Table 1:** Characteristics of the included studies

Authors [reference]	Year	Country	Number of participants	Duration of intervention	Frequency of SMS	Content of SMS	Measure of adherence
Owolabi Eo, et al. [28]	2020	South Africa	I = 108 C = 108	6 months	At an agreed time of the day	Reminder, motivational and support messages, and advice on lifestyle behaviors	MMAS-8
Arora S, et al. [17]	2014	USA	I = 64 C = 64	Six months	Two messages daily for six months	Reminder, motivational, expert opinion, and healthy food choices	MMAS-8
Abaza H et al., [25]	2017	Egypt	I = 34 C = 39	Three months	Daily for three months and four times per week	Reminder, Educational, interventional‚ and lifestyle messages	MMAS-4
Gautier J-F et al., [26]	2021	France	I = 170 C = 136	Three months	Daily for three months	Reminder and importance of adopting healthy behaviors	MMAS-8
Kleinman NJ et al. [27]	2017	India	I = 44 C = 46	Six months	Not specified	Reminders and clinical information	Self-reported
Sugita H et al. [29]	2017	Japan	I = 21 C = 20	Six months	Twice a week for six months	Healthy life-related text messages and the reminder message	MMAS-8
Vervloet M, et al. [15]	2012	Netherlands	I = 56 C = 48	Six months	Daily for six months	SMS reminders	RTMM
Vervloet M et al., [16]	2014	Netherlands	I = 56 C = 57	Two years/24 months	Not specified	SMS reminders	Medication refill adherence
Adikusuma W et al., [30]	2017	Indonesia	I = 25 C = 25	Three months	Once daily for three months	SMS reminders	MMAS-8

### Risk of bias in included studies

**Fig. 2:**
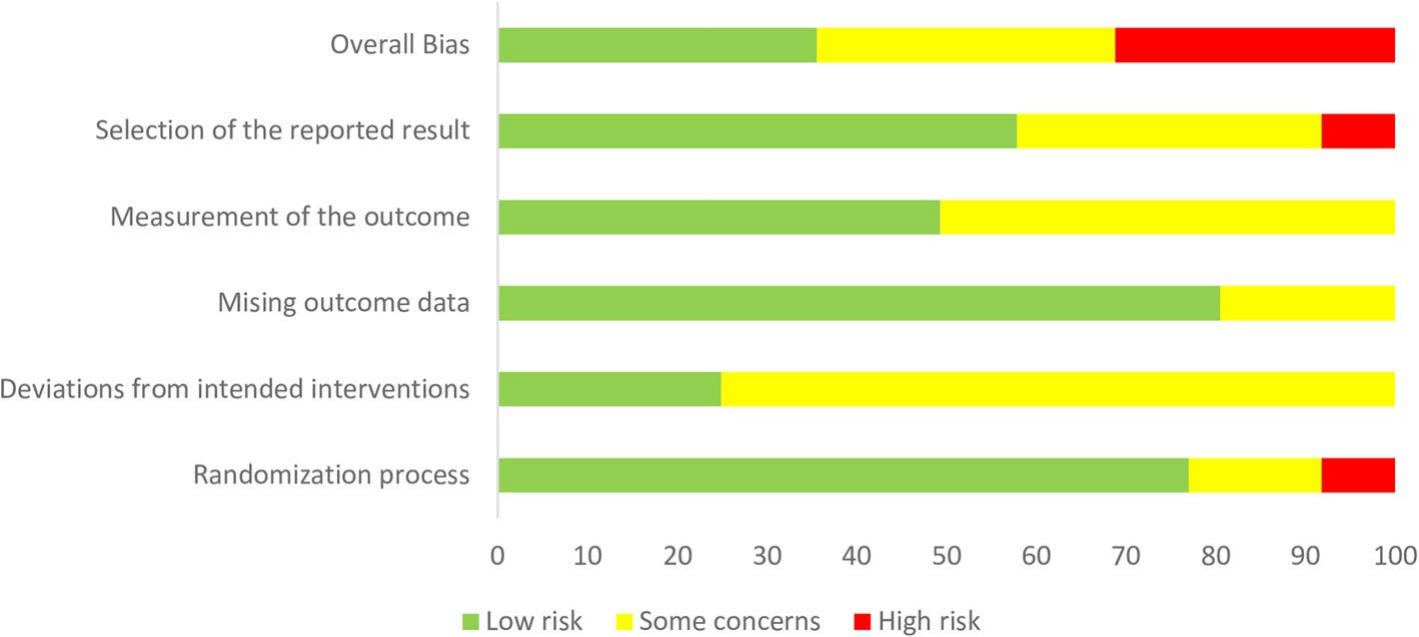
Risk of bias graph: review authors' judgments about each risk of bias as percentages across all included studies

**Fig. 3:**
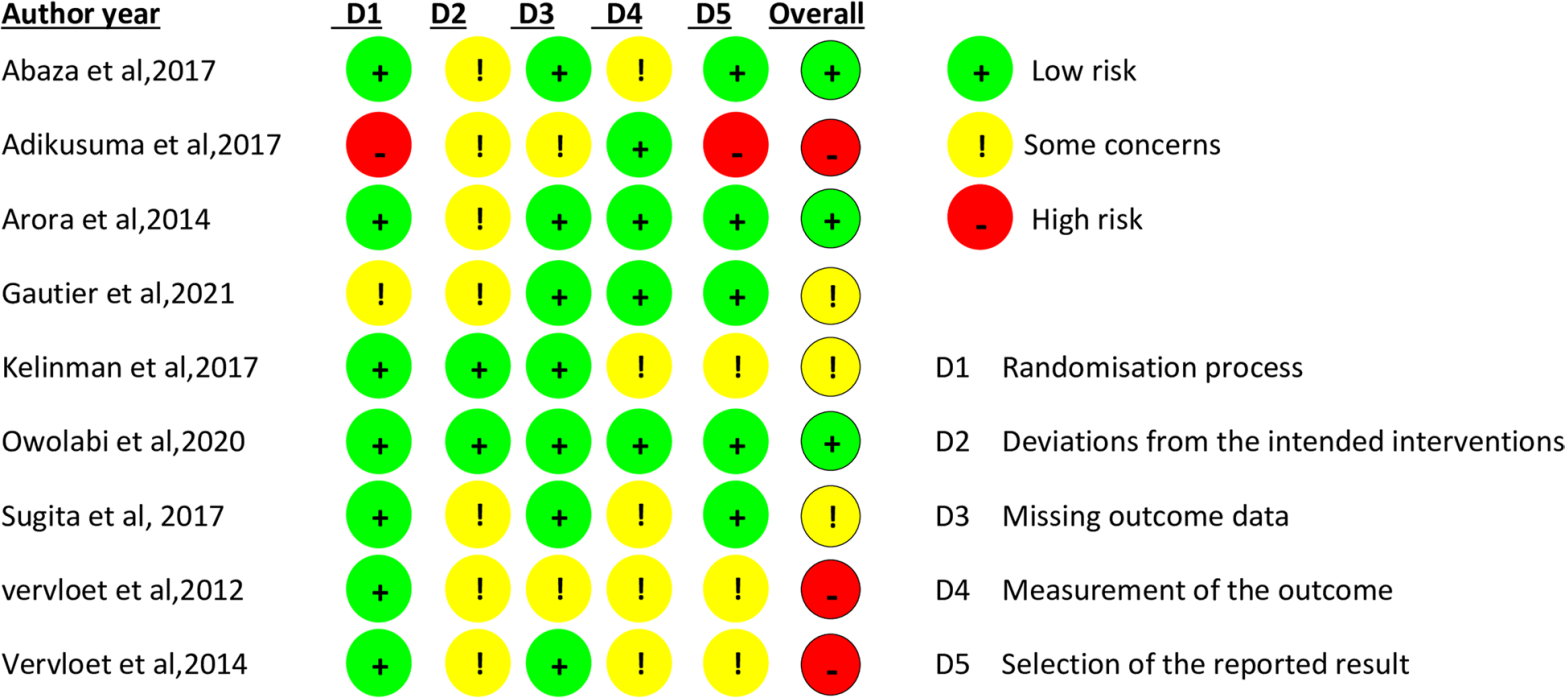
Risk of bias summary: review authors' judgments about each risk of bias in each included study

The assessment of the risk of bias in included studies is shown in Figs. 2 and 3. The methodological quality assessments of RCTs were conducted according to the revised Cochrane risk-of-bias tool for randomized trials (RoB 2) [21]. Eight out of nine studies [15–17, 25–29] designated random sequence generation, resulting in a low bias risk. Allocation concealment was described in 2 of the included studies [27, 28], which results in a low risk of bias due to the presence of allocation concealment. Five studies [15, 17, 25, 27, 28] described blinding participants and personnel as having a low risk of bias.

### The effect of mobile phone text messages on medication adherence

**Fig. 4:**
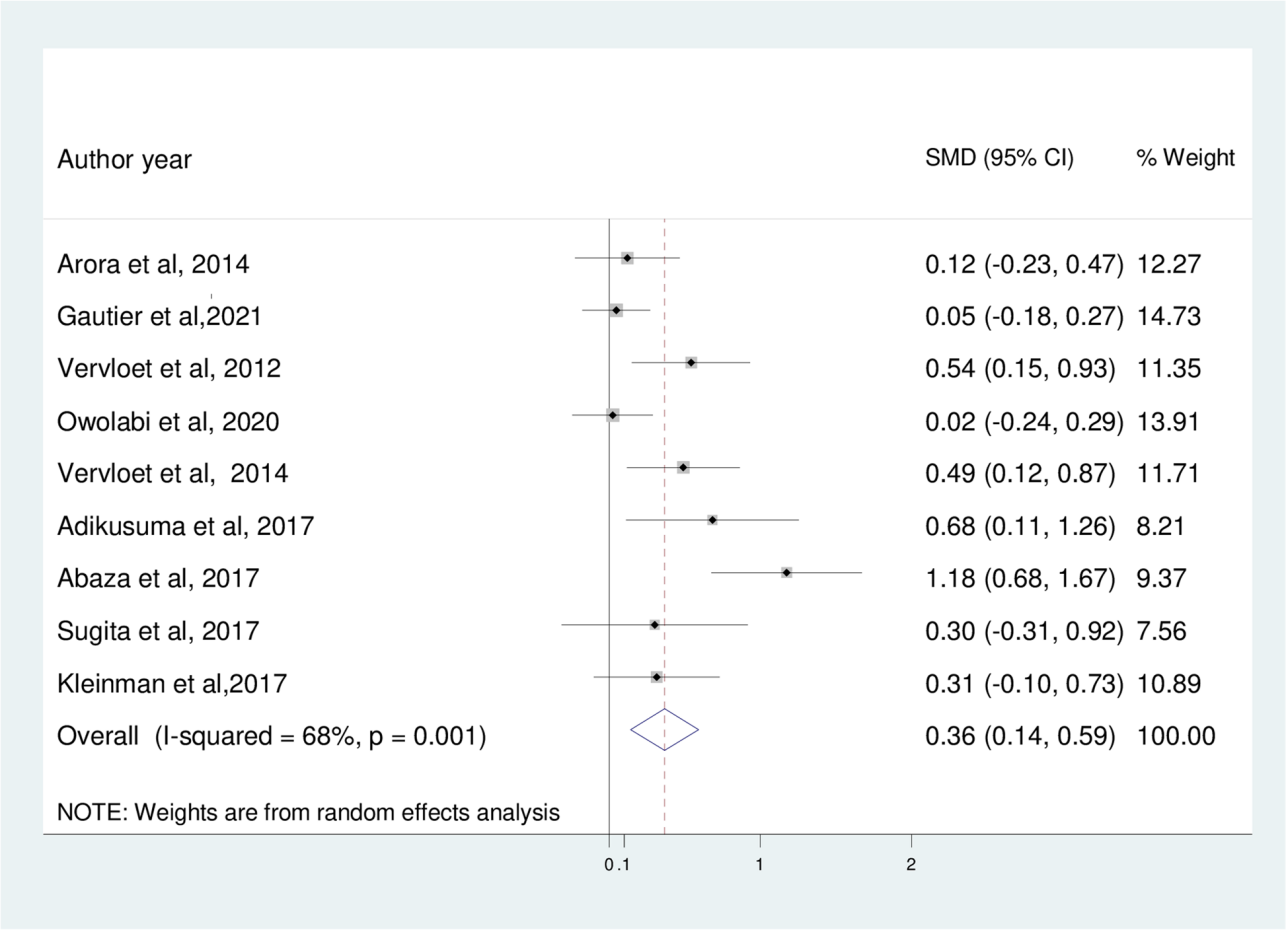
The effect of mobile phone text message intervention to improve medication adherence

In the current meta-analysis, the pooled effect size of mobile phone text message intervention significantly improved medication adherence level (SMD: 0.36; 95%CI; 0.14, 0.59) compared to usual care groups among patients with type 2 diabetes mellitus (Fig. 4). However, the I^2^ statistics among the studies was 68%, indicating a moderate risk of heterogeneity.

### Subgroup analysis

**Fig. 5:**
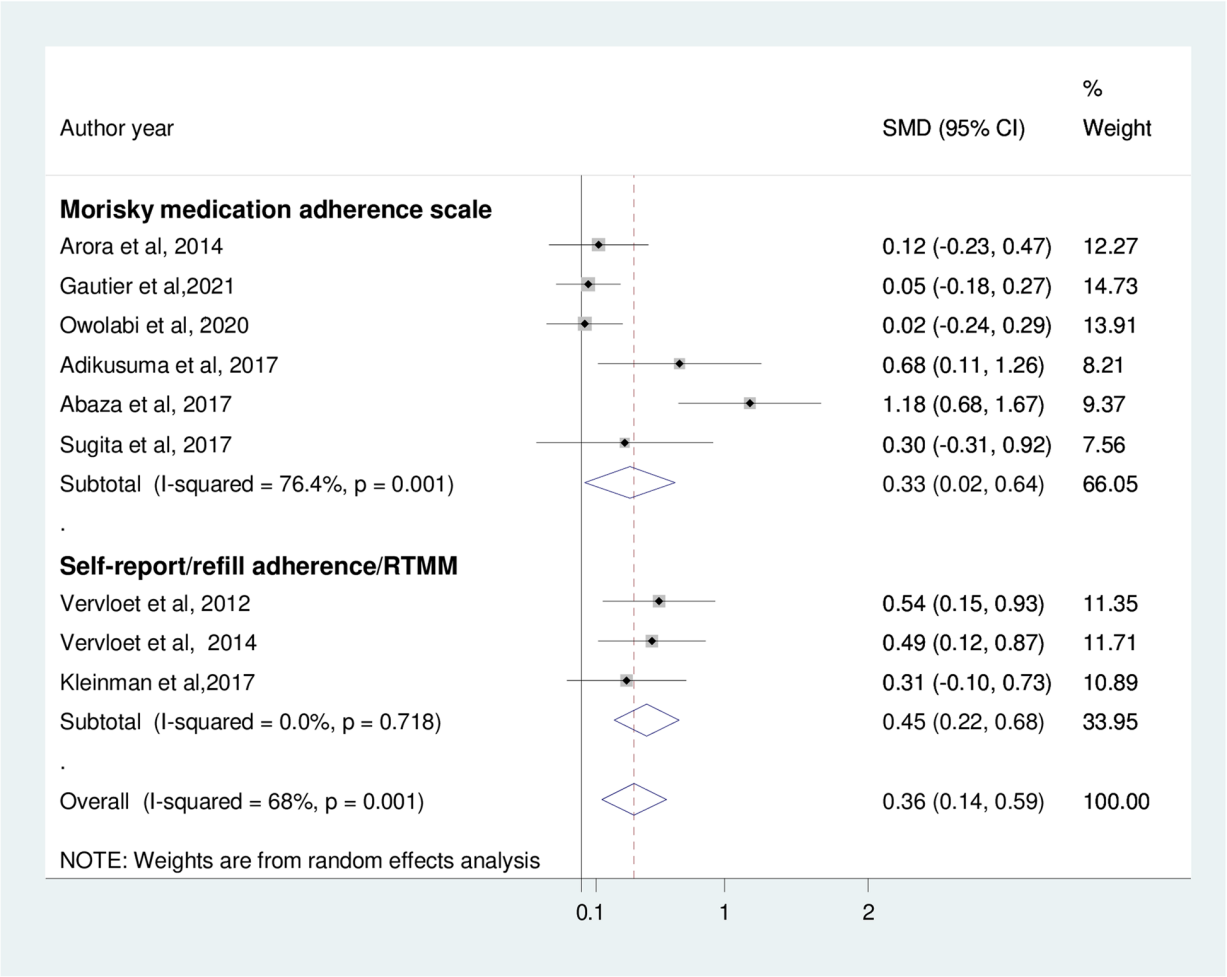
Subgroup analysis based on measures of medication adherence

**Fig. 6:**
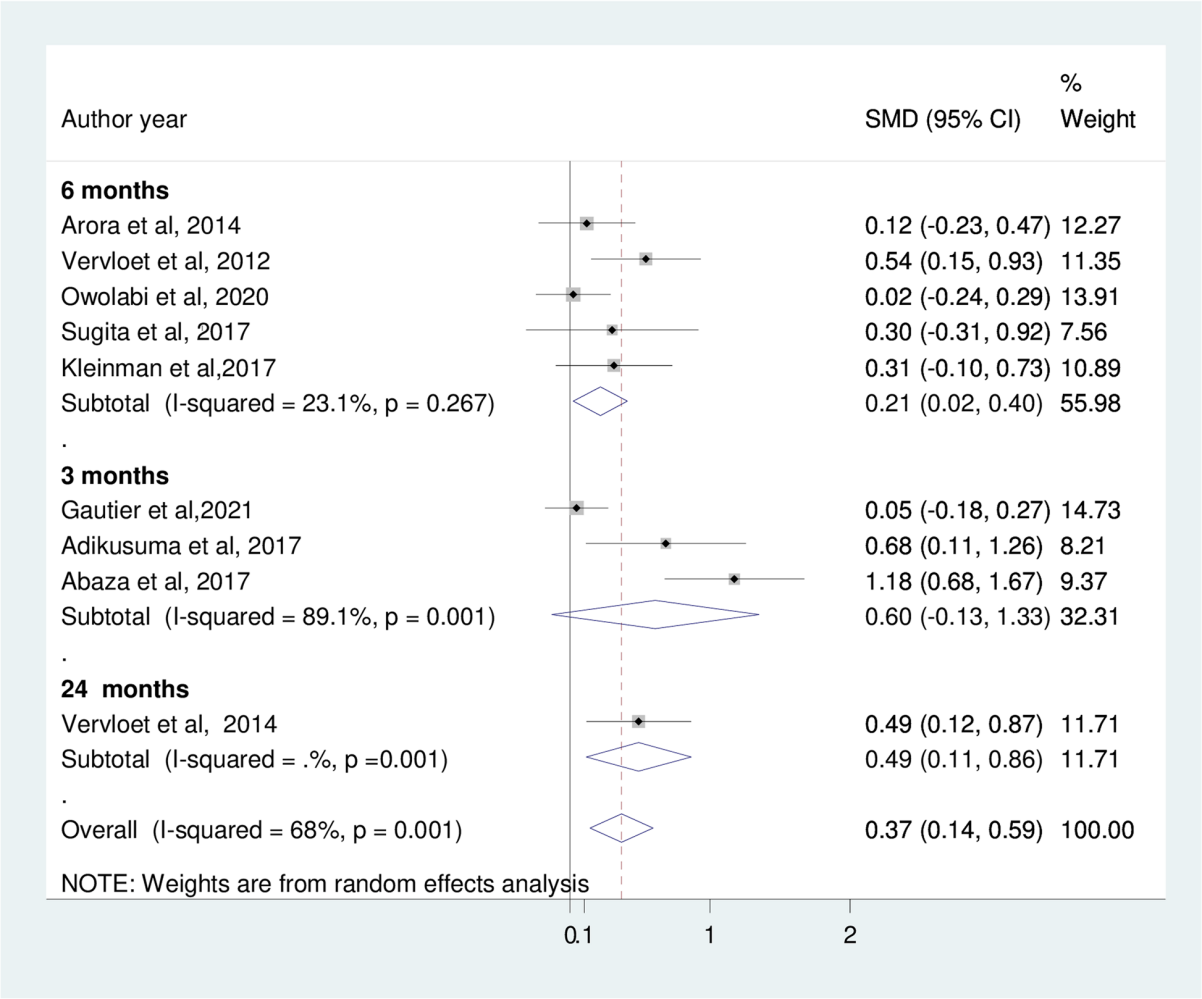
Subgroup analysis based on the duration of intervention

The results of subgroup analyses using the measures of medication adherence show that both the Morisky medication adherence scale (SMD: 0.33; 95%CI: 0.02, 0.64) and self-report/refill adherence (SMD: 0.45; 95%CI: 0.22, 0.68) reveals improvement of medication adherence (Fig. 5). In addition, six months and above interventions have demonstrated significant benefits in improving medication adherence among type 2 diabetes patients (Fig. 6).

### Evaluation of publication bias

**Fig. 7:**
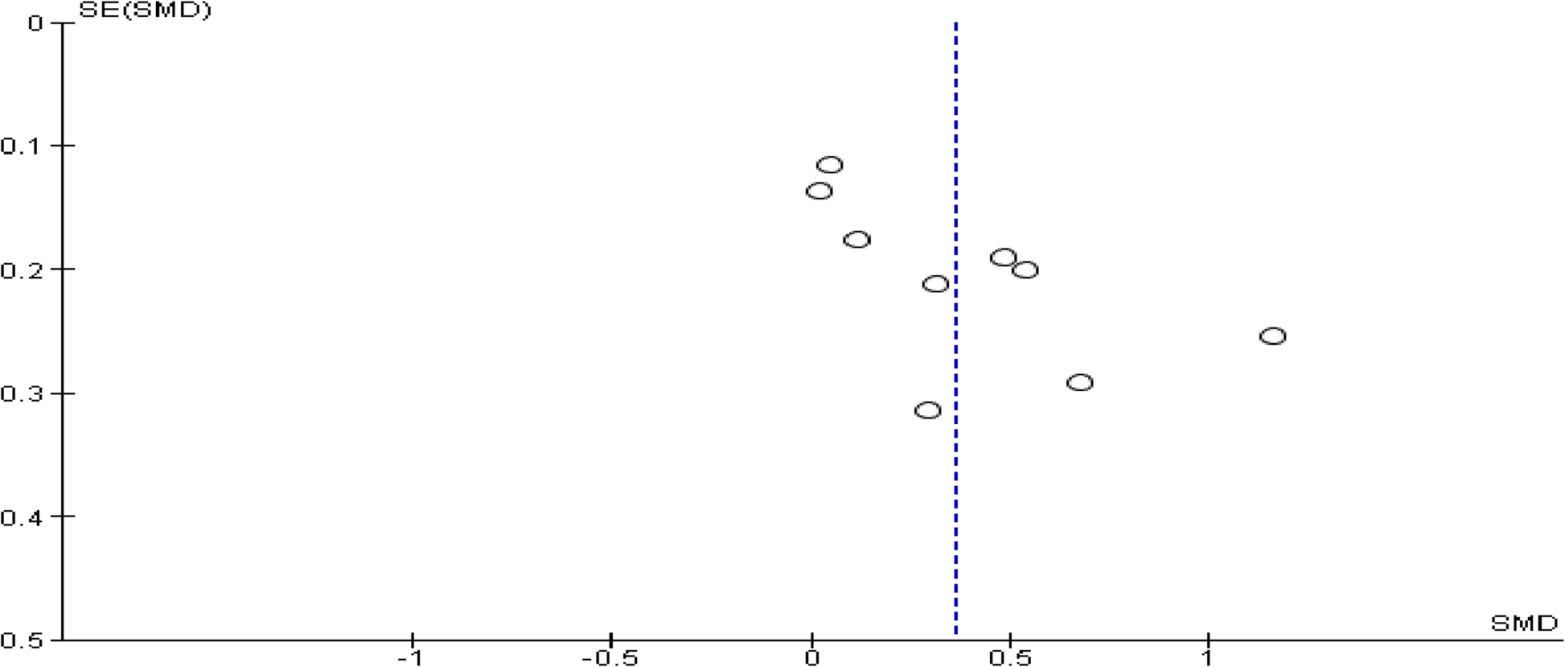
Publication bias among included studies

We constructed a funnel plot and egger’s regression test to investigate potential publication bias. As illustrated in Fig. 7, the funnel plot's visual inspection revealed the studies' symmetrical distribution. Besides, egger’s regression test was (*p* = 0.34), indicating no publication bias.

### The Overall quality of the evidence

The certainty of the evidence was assessed using Cochrane risks of bias together with the five GRADE considerations (study limitations, consistency of effect, imprecision, indirectness, and publication bias). The quality of evidence for medication adherence was found to be moderate evidence; which suggests further studies will increase our confidence in the estimate of effect size (Table 2).

**Table 2:** Summary of findings the effect of mobile phone text message interventions compared to usual care

[mobile phone text message] compared to [usual care] for [medication adherence among T2DM]					
**Patient or population:** Type 2 diabetes mellitus **Setting:** Any healthcare facility **Intervention:** Mobile phone text message **Comparison:** usual care					
**Outcomes**	**№ of participants** **(studies)** **Follow-up**	**Certainty of the evidence** **(GRADE)**	**Relative effect** **(95% CI)**	**Anticipated absolute effects**	
				**The risk with [comparison]**	**Risk difference with [intervention]**
medication adherence assessed with: months follow-up: range three months to 24 months	1121 (9 RCTs)	⨁⨁⨁◯ Moderate^,b^	-	-	SMD **0.36 SD higher** (0.14 higher to 0.59 higher)
*The risk in the intervention group (and its 95% confidence interval) is based on the assumed risk in the comparison group and the **relative effect** of the intervention (and its 95% CI) **CI:** confidence interval; **SMD:** standardized mean difference					
**GRADE Working Group grades of evidence** **High certainty:** we are very confident that the true effect lies close to that of the estimate of the effect **Moderate certainty:** we are moderately confident in the effect estimate: the true effect is likely to be close to the estimate of the effect, but there is a possibility that it is substantially different **Low certainty:** our confidence in the effect estimate is limited: the true effect may differ substantially from the effect's estimate **Very low certainty:** we need more confidence in the effect estimate: the true effect is likely to be substantially different from the estimate of effect					

**Explanations**

a. moderate level of heterogeneity

b. Differences in outcomes measures (e.g. outcome measured at three months vs. at 24 months)

## Discussion

We found nine randomized control trials that evaluated the effect of text messaging interventions on medication adherence in participants with T2DM. Studies generally have a low risk of bias regarding random sequence generation and incomplete outcome data. Allocation concealment was described in 2 of the included studies [27, 28], resulting in a low risk of bias due to allocation concealment. Five studies [15, 17, 25, 27, 28] described blinding participants and personnel as having a low risk of bias. Outcome assessors were reported to have a low bias risk in three studies [17, 25, 28]. Most of the studies [16, 17, 25, 27–30] described a low risk of bias for incomplete outcome data using an intent-to-treat analysis. In the current review, two of the nine included studies [15, 30] demonstrate some concern about the possible risk-of-bias judgments to selective reporting.

Authors found that mobile phone text messaging interventions had a favourable effect on medication adherence in patients with T2DM, with significant improvement in medication adherence by 0.36% when nine studies were pooled together in a meta-analysis. The body of evidence relating to the effect of mobile phone-based interventions on anti-diabetic medication adherence was a moderate level of evidence. Pooled analysis of nine trials showed a paramount benefit for medication adherence for interventions delivered through educational and motivational text messages about T2DM. The studies were conducted in different areas of the globe and provided reasonable confidence in the applicability of results across settings.

Our finding of evidence for the effects of mobile phone text message-delivered interventions to increase adherence to medication prescribed for T2DM is consistent with one meta-analysis determining the effectiveness of short message service intervention to improve glycated hemoglobin control and medication adherence in T2DM [31]. Similarly, this finding is consistent with a systematic review of diabetic self-management education and support apps interventions to improve medication adherence from slight to moderate effects [32]. However, one systematic review examining RCTs of monitoring and messaging interventions targeting medication adherence to managing type 2 diabetes found no evidence of improvements in medication adherence in their pooled meta-analyses of five trials [33].

Our finding showed that pooled analyses of interventions delivered by text messaging indicated moderate benefits with statistical significance. Our findings were consistent with the findings from trials using a short message system alone targeting adherence to cardiovascular disease medication, which also report small benefits of mobile phone text interventions [34]. Medication adherence is essential for maximizing the effectiveness of pharmaceutical therapy [35]. It is known that improved medication adherence may have also influenced glycemic control [36]. Nevertheless, future research must confirm the association between medication adherence and HbA1c levels. The distinct advantages of text messaging over other interventions are simplicity and ease of administration, often in an automated fashion using a computerized program [37].

The finding of the subgroup analysis showed that medication adherence improved significantly after six months of intervention. This implies that we are unable to suggest the effectiveness of mobile phone-based interventions for short-term adherence to medication prescribed for T2DM. Only one study found the longest intervention period for medication adherence out of the studies was 24 months; therefore, it is difficult to predict the effect of longer interventions. Further research is required to shed more light on the long-term effect of mobile phone text message interventions on medication adherence.

This is the first systematic review and meta-analysis of the effectiveness of mobile phone text messaging in improving medication adherence among T2DM. Future studies would consider the following limitations. First, we have limited papers to those written in English. Second, the authors did not include individuals with type 1 diabetes, gestational diabetes mellitus, or prediabetes. Third, the long-term effects of text messaging on medication adherence could not be examined due to the short duration of the trials included in the analysis. Fourth, in some studies, the level of medication adherence was self-reported; this would raise the possibility of reporter bias. Fifth, it is likely that these interventions would require adaptation for different settings, and it needs to be clarified what behavior change techniques, or combinations of them, are effective. Finally, many confounding factors might influence the patients' adherence that we are not maintaining in this review, such as age, sex, duration of diabetes, educational level, cultural background, and socioeconomic class.

## Conclusion

Our meta-analysis pooled results of nine randomized controlled trials demonstrated the favourable impact of mobile phone text messaging on medication adherence in patients with T2DM. Therefore, text messaging, when delivered in addition to usual care, has the potential to produce significant improvements in medication adherence.

### Implication for clinical practice

Our review examined the effectiveness of mobile phone text message reminders/intervention and found an excellent positive effect on T2DM medication adherence. These findings can have important implications for everyday practice and policy, particularly during pandemic times or when healthcare resources are stretched, as novel methods of healthcare delivery in remote and contactless ways become increasingly necessary. Mobile phone text message interventions hold promise in improving adherence. However, implementation and long‐term sustainability need to be considered.

Our findings suggest possible effectiveness, but our review did not find any studies that reported affordability/cost‐effectiveness or how interventions may impact equitable access to T2DM treatment and management. However, the evidence demonstrated from previous analyses of mobile-based interventions in other fields showed inexpensive once systems are set up. Furthermore, compared to other digital interventions, mobile phones are widely prevalent in modern society; short text message interventions are likely more affordable and easily accessible to the general population, including remote and rural populations, as mobile phones are ubiquitous across low‐, middle‐, and high‐income countries. In addition, mobile phone text message interventions may reduce access barriers such as lack of transportation, inability to get time off work, medical consultation costs, and finances.

Mobile phone interventions should be considered part of the more comprehensive health service delivery. Future mobile phone‐based interventions should consider the context and needs of the population, for example, literacy, phone use, use of other services, and what behavior change techniques delivered by mobile phones are likely to be effective. For example, it is not given that people deemed illiterate in a classic reading and writing context cannot benefit from text message interventions as they may still be capable of understanding text messages. Hence, policymakers and other concerned bodies should design appropriate implementation guidelines to improve medication adherence using mobile phone text messages as a care package.

### Implication for research

More extensive trials with extended follow-up periods using theory-based interventions are required to improve current evidence. In addition, further study is required on the feasibility, cost-effectiveness, and acceptability of mobile phone text message interventions. Finally, future studies need to determine the features of text message interventions that improve success, appropriate patient populations, sustained effects, and influences on clinical outcomes.

## Supplementary Information

**Additional file 1.**

## Data Availability

All data generated or analyzed during this study are included in this published article [and its supplementary information files].

## Abbreviations

DM: Diabetes Mellitus
GRADE: Grading of Recommendations Assessment, Development, and Evaluation
RCT: Randomized control trial
SMD: Standard Mean difference
T2DM: Type 2 Diabetes Mellitus
