# Supplementary material for: What is the effect of mobile phone text message reminders on medication adherence among adult type 2 diabetes mellitus patients: a systematic review and meta-analysis of randomized controlled trials
Source: BMC Endocr Disord. 2023 Jan 19;23:18. doi: 10.1186/s12902-023-01268-8 (PMC9850787; doi:10.1186/s12902-023-01268-8)
Supplement: Supplementary file 1 — Additional file 1. [file 12902_2023_1268_MOESM1_ESM.docx]

| Supplementary Table 1. PubMed search history | | |
| --- | --- | --- |
| Search | Search terms | Hits |
| #1 | Type 2 diabetes[tw] OR Type 2 diabetes mellitus[tw] OR T2DM[tw] OR insulin non dependent diabetes [tw] | 209,740 |
| #2 | Phone message [tw] OR short message service [tw] OR phone call [tw] OR mobile phone message [tw] OR text message [tw] | 4,759 |
| #3 | Adherence [tw] OR compliance [tw] OR patient compliance[tw] OR Medication adherence[tw] | 326,182 |
| #4 | #1 AND #2 AND #3 | 204,143 |
| #5 | #4; Limits: studies done with Humans, English language, full text, RCT, and publication year (2000 to 2022) | 237 |
